# The Role of Human Herpesvirus 6 Infection in Alzheimer’s Disease Pathogenicity—A Theoretical Mosaic

**DOI:** 10.3390/jcm11113061

**Published:** 2022-05-29

**Authors:** Constantin Romanescu, Thomas Gabriel Schreiner, Ilya Mukovozov

**Affiliations:** 1 Clinical Section IV, “St. Parascheva” Infectious Disease Hospital, 700116 Iași, Romania; 2 Faculty of Medicine, University of Medicine and Pharmacy “Gr. T. Popa”, 700115 Iasi, Romania; 3 Faculty of Medicine, University of Medicine and Pharmacy “Carol Davila”, 050474 Bucharest, Romania; 4 Department of Electrical Measurements and Materials, Faculty of Electrical Engineering and Information Technology, Gheorghe Asachi Technical University of Iasi, 21–23 Professor Dimitrie Mangeron Blvd.,700050 Iasi, Romania; 5 Department of Dermatology and Skin Science, University of British Columbia, Vancouver, BC V6T 1Z4, Canada; mukovoi@gmail.com

**Keywords:** HHV-6A, HHV-6B, Alzheimer’s disease, amyloid beta, miRNA 155, autophagy

## Abstract

Alzheimer’s disease (AD), a neurodegenerative disorder generally affecting older adults, is the most common form of dementia worldwide. The disease is marked by severe cognitive and psychiatric decline and has dramatic personal and social consequences. Considerable time and resources are dedicated to the pursuit of a better understanding of disease mechanisms; however, the ultimate goal of obtaining a viable treatment option remains elusive. Neurodegenerative disease as an outcome of gene–environment interaction is a notion widely accepted today; a clear understanding of how external factors are involved in disease pathogenesis is missing, however. In the case of AD, significant effort has been invested in the study of viral pathogens and their role in disease mechanisms. The current scoping review focuses on the purported role HHV-6 plays in AD pathogenesis. First, early studies demonstrating evidence of HHV-6 cantonment in either post-mortem AD brain specimens or in peripheral blood samples of living AD patients are reviewed. Next, selected examples of possible mechanisms whereby viral infection can directly or indirectly contribute to AD pathogenesis are presented, such as autophagy dysregulation, the interaction between miR155 and HHV-6, and amyloid-beta as an antimicrobial peptide. Finally, closely related topics such as HHV-6 penetration in the CNS, HHV-6 involvement in neuroinflammation, and a brief discussion on HHV-6 epigenetics are examined.

## 1. Introduction

Human herpesviruses 6 (HHV-6), including human beta-herpesvirus 6A (HHV-6A) and human beta-herpesvirus 6B (HHV-6B), belong to the Herpesviridae family of DNA viruses that infect humans and other animals. HHV-6 was originally detected in the context of the acquired immunodeficiency syndrome (AIDS) epidemic of the 1980s in the human immunodeficiency virus (HIV) seropositive population of North America [1]. Presently, HHV-6 continues to be studied in various areas of the medical sciences, with a considerable body of work reporting an association between HHV-6 and various neurological pathologies, the most relevant being multiple sclerosis (MS) [2], epilepsy [3], and Alzheimer’s disease (AD) [4]. Throughout the course of the last two decades, new evidence concerning mechanisms by which HHV-6 is involved in producing both acute and chronic infections has emerged. Considered to be extremely widespread with close to 100% infection rates among the general population in some parts of the world [5], HHV-6 has been studied extensively in the infant population. In children under 3 years old, HHV-6 infection is characterized by high fevers and specific skin lesions called exanthema subitum, also known as roseola infantum [6], with the HHV-6B subtype being most often cited as the cause of infection among the pediatric population in Europe and North America [7].

Alzheimer’s disease (AD), a neurodegenerative disorder associated with severe cognitive and psychiatric decline, affecting mostly older adults, is the most common form of dementia worldwide [8]. Despite being one of the most studied non-communicable diseases [9], satisfactory treatment options for AD are still not available, to the detriment of the patient and society alike.

Years of intense research devoted to the understanding of AD pathophysiology has yielded evidence suggesting the existence of a multifactorial etiology in disease onset and progression. Some of the key phenomena involved include the misfolding of amyloid beta (Aβ) [10] and Tau proteins [11], chronic neuroinflammation [12], and oxidative stress [13].

Neurodegenerative disease as an outcome of gene–environment interactions is a notion widely accepted in the present day; however, a clear understanding of how external factors are involved in pathogenesis is missing. In the case of AD, significant effort has been invested in the study of viral pathogens such as human herpesvirus-1 (HSV-1), cytomegalovirus (CMV), HHV-6, and hepatitis C virus (HCV), and their role in disease mechanisms [14]. In light of the crisis defined by the high incidence and prevalence rates of AD combined with inadequate management, and an incomplete understanding of the disease, the viral concept of disease is gaining traction. Some of the early research investigating the viral etiology of AD dates back approximately 20 years ago, with reports of HSV-1 presence in diseased brain samples constituting a risk factor in carriers of the apoE-ε4 allele of the apolipoprotein E (APOE) gene [15]. Because many of the initial studies incidentally identified the existence of other viruses (aside from HSV-1) in significantly higher quantities in AD specimens versus non-dementia controls, such as those aforementioned, the scope of research in this area has since broadened significantly. Going beyond demonstrating viral presence in brain tissue or in peripheral blood samples, newer research is aimed at uncovering how viral infection can directly or indirectly interact with host biology to play a role in disease formation. Research has thus far generated optimism—with compelling evidence presented in favor of the viral concept of Alzheimer’s disease; however, as contradictory findings emerge to temper the debate, many questions remain unanswered. In this context, the aim of this article was to present the landscape surrounding the viral concept of AD and, more specifically, the role HHV-6 is believed to play in AD pathogenesis.

## 2. Methodology

Given the relatively limited data on the involvement of HHV-6 in AD pathogenicity, the existing literature was considered in the form of a scoping review in order to create a broad picture of both the arguments in favor of the viral hypothesis and those against it. A systematic search was conducted in three online databases, including PubMed/Medline, Embase, and Google Scholar, by combining the search terms “HHV-6”, “HHV-6A”, “HHV-6B”, “Human herpesvirus”, “Alzheimer’s disease”, “Alzheimer’s dementia”, and “dementia”. Original, peer-reviewed, English language studies published between 2000 and 2022 were included. After the screening process, 20 relevant papers were selected for the current scoping review. The search strategy and the final results are outlined in Table 1.

## 3. Evidence of HHV-6 Infection and Alzheimer’s Disease

Some of the first studies to investigate an association between virus and AD sought to demonstrate the presence of HHV-6 in either post-mortem AD brain specimens or in blood samples of living AD patients. Today, as new evidence amasses in this expanding area of research, the results appear to be increasingly mixed and contradictory. A brief scan of the existing literature will reveal the heterogeneity of the results. Table 2 outlines the selected publications reporting results that present compelling arguments both in favor of a significant association between HHV-6 and AD pathogenesis, and against a significant link, with some even denying an important viral presence in the brain samples of AD patients, calling into question the methodologies and techniques employed by some of their colleagues.

**Table 2:** The most relevant research on the HHV-6 infection—AD association—pro vs. contra studies.

Study Cohort, *n* (Controls)	Study Design	Main Results	Reference
**Research reporting the possible role of HHV-6 in AD pathogenesis**			
98 AD (no controls)	Materials: whole blood samples Method: genotyping kits for KIR and HLA alleles Research question: KIR/HLA genetic background in AD	KIR2DS2/KIR2DL2/C1 correlated with patients with lower MMSE score Indirect marker for increased susceptibility to HHV-6A infection	Rizzo et al., 2019 [16]
643 AD (no controls)	Materials: brains from AD patients Method: functional genomic analysis, a multiscale network of LOAD-associated virome Research question: pathogenic HHV-6 regulation of molecular, clinical, and neuropathological networks	Increased HHV-6A from subjects with AD compared with controls	Readhead et al., 2018 [17]
158 AD (228 controls)	Materials: Peripheral blood leukocyte samples Method: Single nucleotide polymorphism detection, genotyping Research question: Specific gene mutations associated with factors regulating antiviral response in AD patients	HHV-6 DNA is (statistically significant) more frequently encountered in AD groups vs. controls Overexpression of IL-28B TT carriers in AD patients Med23 and IRF7 GG genotypes correlated with HHV-6 risk for AD	Licastro et al., 2015 [18]
93 AD (164 ND)	Materials: Peripheral blood leukocyte samples Method: qPCR, genotyping Research question: HHV-6 presence in peripheral blood of AD patients	Significantly increased positivity of HHV-6 in peripheral blood leukocyte samples and brain tissue in AD patients	Carbone et al., 2014 [19]
27 AD (13 controls)	Materials: CSF and serum samples Method: ELISA, PCR Research question: Assessment of the immune response to HHV-6 in AD patients via the detection of intrathecal antibodies	Detectable intrathecal antibody synthesis to HHV-6 in AD patients (in low percentage) versus negative controls	Wozniak et al., 2005 [20]
50 AD (35 controls)	Materials: Frozen postmortem brains Method: PCR Research question: HHV-6 detection in AD brain specimens	HHV-6 is present in the brain of a far higher proportion of AD patients than of age-matched controls	Lin et al., 2002 [15]
**Research refuting HHV-6 involvement in AD pathogenesis**			
575 definite AD (341 ND)	Materials: 3 independent AD cohorts Method: RNA-seq, PCR Research question: Screening for pathogens (including 118 human viruses) in AD patients	Little specificity of HHV-6 to AD brains over controls by both RNA-Seq and droplet digital PCR methods (no differences in viral detection between the two groups)	Allnutt et al., 2020 [21]
602 AD (no controls)	Materials: Brain samples Method: KrakenUniq (highly sensitive method) Research question: Detection of extremely low HHV-6 read counts in AD brains	Identification via KrakenUniq of HHV-6A reads in only 2 out of the top 15 samples sorted by reported HHV-6A abundance	Chorlton et al., 2020 [22]
50 AD (52 ND)	Materials: Blood samples Method: PCR, multiplex immunoassay Research question: Analysis of IgG reactivity toward several viruses in AD patients	HHV-6 IgG reactivity was significantly lower in AD compared to controls	Westman et al., 2017 [23]
59 AD, 60 aMCI (61 controls)	Materials: Whole blood and serum samples Method: ELISA, MRI, and genotyping Research question: The analysis of HHV-6-specific humoral immunity in AD patients	HHV-6 seroprevalence, antibody titers, and avidity were similar in all three groups	Agostini et al., 2016 [24]
34 AD (40 controls)	Materials: Brain specimens Method: PCR Research question: Detection in brain specimens for HHV-6 DNA	No significant difference for HHV-6 DNA in AD groups compared to the control group HHV-6 is no additional risk factor for AD	Hemling et al., 2003 [25]

Abbreviations used in Table 2: AD—Alzheimer’s disease; ND—non-dementia; HHV-6—human herpesvirus 6; aMCI: amnestic mild cognitive impairment; MMSE: mini mental status exam; HLA—human leukocyte antigen; LOAD—late-onset Alzheimer’s disease; PCR—polymerase chain reaction; MRI—magnetic resonance imaging; ELISA—Enzyme-linked immunosorbent assay; and KIR—killer immunoglobuline receptors.

The earliest study in our review to suggest a link between HHV-6 and AD is based on the discovery of significantly increased viral load in the brain specimens of AD patients compared to non-dementia (ND) controls, with the authors of the study reporting HHV-6 DNA presence in 70% of postmortem AD brain specimens (frontal and temporal lobes) compared to 40% age-matched controls [15]. A more recent study by Carbone et al. [19] discovered HHV-6 positivity in up to 17% of AD patients’ brains, compared to 4% of controls, ascribing the lower percentage vis-a-vis the aforementioned study to a difference in detection methods. Moreover, the research team observed significantly higher HHV-6 load in peripheral blood lymphocyte (PBL) samples from patients with AD, compared to the control group in their study, with serial follow-up at 5 years confirming higher peripheral HHV-6 uptake in patients who had developed clinical symptoms of AD. The relationship between HHV-6 in PBL samples and AD is due to two important characteristics common to the human herpesviruses, namely, their ability to cause latent infection by circumventing the host’s immune system and their tropism for nervous tissue. Going beyond characteristics intrinsic to the virus, Licastro et al. [18] report that individuals who are carriers of the GG genotype of the mediator complex 23 (med23) gene, which is involved in the antiviral immune response, may be more prone to developing AD in response to latent, sub-clinical infection with HHV-6. More specifically, this particular genotype is associated with increased HHV-6 positivity in PBL samples of AD patients. It appears that, at least partially, an inadequate immune response can explain the link between HHV-6 in PBL samples and AD (see below Section 5.2 for further discussion). That some individuals with HHV-6 latent infection develop AD and others do not is an example of the complex interplay between genes and environment, where traits inherent to both the virus and the individual combine to generate a specific disease outcome.

As is often the case in science, however, novel concepts and theories are not left unchallenged for long. Several studies present evidence that challenges some of the findings in support of the viral hypothesis. For example, Hemling et al., 2003 [25], report no difference between HHV-6 DNA load in AD patients compared to the controls. Recently, the study conducted by Allnutt et al. [21], on a much larger cohort (575 definite AD) and using modern detecting techniques such as RNA-Seq and droplet digital PCR, found no differences in HHV-6 detection between the AD group and the control group. There are also uncertainties regarding the reactivation of the dormant virus at the brain parenchyma level. The work of Wozniak et al. [20] revealed there is no significant difference between the controls and the intrathecal antibody synthesis in response to HHV-6 in AD patients. The researchers suggest this could mean either no acute infection had occurred, the reactivation of HHV-6 had not taken place at the CNS level, or the immune response had been weak. These are only speculations as the complete immunological reactivity is not completely understood. An aberrant immune response was also suggested by a recent study conducted by Westman et al. [23]. The research team reported significantly lower HHV-6 IgG reactivity in AD patients compared to the controls. Other similar studies, conducted on individuals with AD, amnestic mild cognitive impairment patients, and healthy controls, showed that HHV-6 seroprevalence, antibody titers, and avidity were similar in all the three mentioned groups and cannot be considered proof of the association between HHV-6 infection and AD [24]. In addition, no link can be established between HHV-6 (antibody titer or avidity) and brain atrophy as revealed by MRI.

The debate surrounding the plausibility of HHV-6 infection in AD etiology is ongoing as contradicting evidence continues to surface. On the one hand, some studies point towards a potential impact of herpesviruses in dementia onset, with additional elements such as HLA subsets playing significant roles. Rizzo et al. [16] found a correlation between KIR2DS2/KIR2DL2/C1, a lower MMSE score (representative for AD), and an increased susceptibility to HHV-6A infection. On the other hand, a very recent study on AD murine models (5XFAD mice) did not support the role of murine or human roseola viruses in the development of AD-specific Aβ senile plaque formation [26]. The authors do, however, demonstrate HHV-6 involvement in generating and maintaining neuroinflammation, but the fact that the pathological formation of Aβ agglomeration is not achieved is further evidence that there are other mechanisms—still incompletely elucidated—involved in this complex neurodegenerative process.

Readhead et al. [17] conducted a multiscale analysis on a large cohort of late-onset AD postmortem specimens addressing multiple issues related to molecular, genetic, and clinical aspects of the HHV-6—AD association. According to the authors, an increased HHV-6A level was found in the brains of AD subjects compared with the controls. The importance of this study also lies in the fact that several pathophysiological aspects of HHV-6A’s impact on AD are reviewed, opening new research directions. However, the results obtained by Readhead et al. [17] were subsequently questioned by researchers in the following years. Chorlton et al. [22] reanalyzed the specimens belonging to the same cohort using a different detection method. The results were discordant, and the much lower figures obtained suggested a lack of any relevant correlation between HHV-6 and AD. The sensitivity of the detection method plays an important role, especially when trying to detect extremely low viral loads. Besides currently existing insufficient sensitive methods, another potential limitation in studies focusing on HHV-6 detection is the absence of a standardized protocol or of a specific viral threshold. Moreover, Jeong and Liu [27], after re-running the statistical analyses, concluded that there was a lack of statistical robustness and an imprecise analysis of the datasets in the work of Readhead et al. [17] and that the very low levels of HHV-6 RNA and DNA in AD brains may lead to false interpretations.

Differences in study methodology, combined with individual traits and other still incompletely understood aspects of both HHV-6 chronic infection and AD pathogenesis, could account for the lack of consistency in the above-reported findings.

## 4. Research Directions Investigating HHV-6 Involvement in Alzheimer’s Disease—Selected Examples

**Figure 1:**
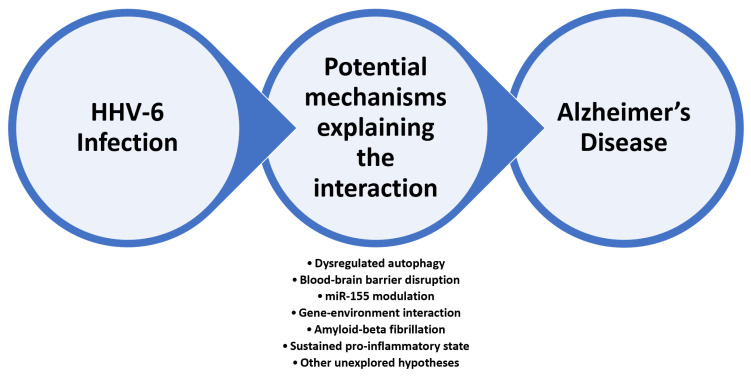
A summary of the mechanisms involved in the interaction between HHV-6 and Alzheimer’s disease.

The authors of the present study selected some of the main research directions investigating the HHV-6–AD relationship for review (Figure 1).

### 4.1. HHV-6 as the Main Deregulator of Autophagy Mechanism at the CNS Level

The autophagy pathway is an important mechanism for intracellular protein degradation. It appears to be involved in certain disease states, such as neurodegenerative pathology, including AD [28]. Within the autophagy mechanism, several structures are involved, the first one being the autophagosome precursor, known as the phagophore. After engulfing a substrate, the phagophore forms edges that converge to form a vesicle—the so-called veritable autophagosome, which subsequently fuses with the lysosome. The entire process is regulated by a series of autophagy-related (ATG) proteins; further details extend beyond the scope of the present article but have been described in recent reviews extensively [29].

Certain disease states, such as Alzheimer’s disease, can interfere with the autophagy pathway at various stages, leading to the impairment of autophagosome and autolysosome formation, lysosomal function, and cargo recognition. Abnormal Aβ accumulation in brain tissue is one of the hallmarks of AD [30]; however, the bidirectional Aβ-autophagy relationship is not completely understood. Recent research reports that a probable cause of AD likely involves impaired the elimination and degradation of Aβ rather than the increased production of Aβ fibrils [31]. Indeed, the autophagy-lysosome pathway, together with the ubiquitin-proteasome and endosome-lysosome pathways, which represent the intracellular degradation of Aβ and an important clearance pathway, are significantly reduced in AD. Several studies in this direction demonstrated that the upregulation of the autophagic pathway may reduce Aβ levels in different body compartments [32]. Other research highlighted, however, the possibility of this pathway promoting Aβ production as the autophagosomes seem to contain amyloid precursor protein (APP) and Presenilin-1 (PS-1), enzymes involved in the formation of Aβ (see amyloidogenic pathway) [33].

The role of genetic influence in AD pathogenesis is an ongoing subject of research, with some mutations already demonstrated to play relevant roles in disease onset and evolution. In this context, it is important to mention the PS-1 mutation, which, besides contributing to the increased production of Aβ, is linked to the downregulation of lysosomal v-ATPase, which results in increased lysosomal pH and reduced autophagosome activity [34].

Romeo et al., 2019 [35] also investigated autophagy dysregulation and reported HHV-6A and HHV-6B as expressing different behaviors in the autophagy process. While HHV-6A was a promotor factor for autophagy, HHV-6B was associated with inhibited autophagy in Molt-3 cells and peripheral blood mononuclear cells (PBMC). The complete picture of the immune response evoked by HHV-6 is unavailable; however, more recent results show that HHV-6 is able to up-regulate the programmed death ligand 1 (PD-L1) on the surface of infected monocytes while simultaneously increasing its extracellular release, thus leading to an impairment of anti-viral immune response [36].

### 4.2. miRNAs as a Valuable Link between HHV-6 Infection and Alzheimer’s Disease

miRNAs are a heterogeneous group of molecules that play a role in both normal physiology and in various neurological [37] and infectious [38] pathologies. miRNAs are found in large quantities in the brain [39], where they modulate neurogenesis and synaptogenesis, along with other neurophysiological functions [40]. In this context, several studies have focused on the roles of different types of miRNAs, especially miRNA 155 (miR-155) in neurodegenerative diseases, including AD [41].

**Figure 2:**
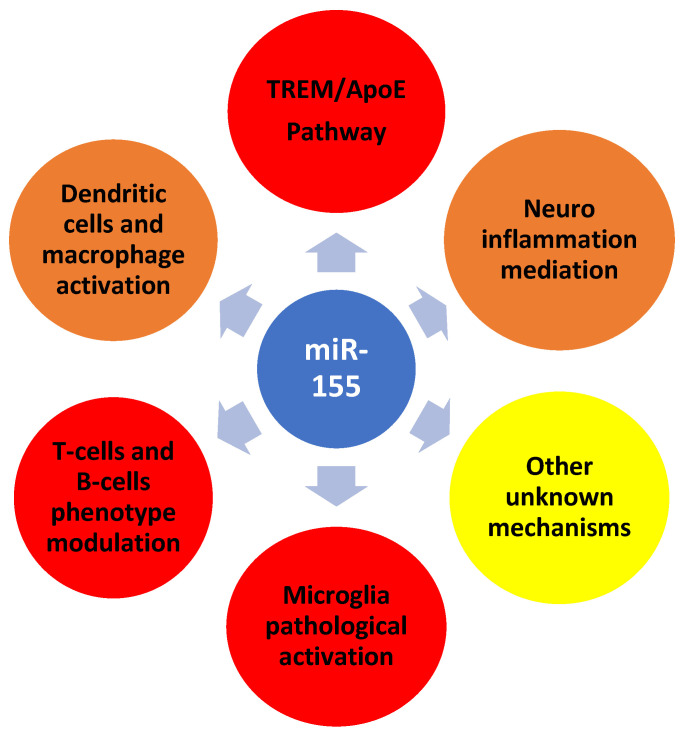
The cellular and molecular pathways/targets explaining the impact of miRNA (focus on miR-155) in Alzheimer’s disease. (Color coding: blue—promoting factor; red—negative impact; orange—negative or positive impact (related to other biological variables); and yellow—unknown).

The miRNA-AD link is complex and is at present being actively researched. It appears miRNA could be of use as a biomarker in early AD detection and in the monitoring of evolution and therapy outcomes in AD patients [42]. Furthermore, miRNA may also be of potential use in therapy as a drug target [43]. These issues are further discussed below in relation to and with an emphasis on HHV-6 infection. Figure 2 summarizes the major findings in miRNA-AD research.

Recent work by Guedes et al. [44] provides insight into the impact of miR-155 dysfunction (mainly upregulation) in AD mouse models and, interestingly, Caselli et al. [45] have shown that HHV-6A suppresses miR155.

Furthermore, Sierksma et al. [46] revealed that 4 miRNAs (including miR155) are relevant to the pathological process in AD, in both animal models such as APP/PSEN1 and Tau22 mice, and in humans. Another study highlighted the impact of other non-coding RNAs (miR-132, miR-129) in specific alterations in the cortical transcriptome that seem to be associated with AD [47].

miR-155 modulates several processes relevant to the onset and evolution of AD. For example, miR-155 mediates neuroinflammatory aspects that subsequently lead to neuronal apoptosis in AD-relevant brain regions. Sun et al. [48] suggested a potential role of miR155 in the activation of microglial cells, which are considered key players in inducing and maintaining neuroinflammation. The authors showed that miR-155 upregulates the production of pro-inflammatory cytokines in microglial cells, with a subsequent negative impact on hippocampal cells. By modulating several apoptosis regulators such as pro-caspase-3 or Bcl-2, miR-155 indirectly leads to neuronal cell death.

Another possible target for miR-155 might be the TREM2-APOE pathway, another relevant pathway involved in the microglial pathological activation encountered in AD [49]. TREM2 and APOE genes mutations, related to microglial and innate immune system dysfunctions, are known to be involved in AD pathogenesis. The TREM2-APOE pathway is one of the main regulators of microglial behavior in neurodegeneration [50]. Moreover, miR-155 is a key factor in the posttranslational regulation of the microglial phenotype that leads to neuronal apoptosis and Aβ plaques [51].

Lastly, miR-155 may exert its influence at the CNS level by modulating the immune system, mainly via T-cell and B-cell regulation. Several studies on adult mice showed the impact of miR-155 on B cell maturation and the expression of important related factors, which subsequently impact IgG1 production [52]. Regarding T-cells, miR-155 seems to control phenotype activation, with a great impact on autoimmune pathology but also bearing relevance to AD. The miR-155 level is essential for T helper cell phenotype induction, with research demonstrating miR-155 deficiency to be correlated with Th2 cell as the dominant phenotype, while miRNA overexpression promotes the Th1 phenotype [53]. In addition, other types of cells considered to have immune functions, such as dendritic cells and macrophages, are fine-tuned by miR-155. For example, miR-155 is considered to target and silence c-Fos expression, thus playing an essential role in dendritic cell maturation [54]. miR-155 regulates macrophage polarization, by inhibiting the M1 pro-inflammatory phenotype, alongside the TGF-β signaling pathway, with a significant negative impact on the restorative and protective role of M2 macrophages [55].

Newer studies suggest miR-155 could contribute to AD pathogenicity via several other pathways. According to Readhead et al. [56], miR-155 is upregulated in critical brain regions related to AD in both APP/PSEN1 mice and humans, the most relevant ones being the dentate gyrus and the CA1 hippocampus. miR-155 was consistently regarded as a molecular mediator of APP/PSEN1 on several cognitive functions, as its deletion seems to have a beneficial impact on learning behavior. miR-155 also has indirect effects that favor AD onset, via changes induced in the transcriptomics and even upregulation of trait loci related to AD high risk.

### 4.3. HHV-6 and Amyloid Beta Fibrillation—Reframing the Amyloid Hypothesis

Bacterial, fungal, and viral infections have been linked to AD mainly via the neuroinflammatory pathway but another potential mechanism related to Aβ was recently proposed. The amyloidogenic hypothesis, although intensely studied, does not seem to explain AD onset alone. HHV-6, alongside other viral pathogens, such as HSV-1, could very well be a missing link in our understanding of excessive Aβ deposition.

In order for senile plaques to be formed, Aβ mono- and oligomers undergo fibrillation to form insoluble brain deposits. One potential trigger for Aβ production and pathological deposition in extracellular brain spaces can be the result of CNS antimicrobial activity sustained by several infections. Eimer et al. [57] observed Aβ oligomers to be actively implicated in neural protection by binding herpesvirus surface glycoproteins. In this manner, the pathological Aβ aggregation is also sustained and increased, at least as results from research on 5xFAD mice and human neuron cell cultures suggest. Thus, Aβ is not simply an endogenous by-product without function but a relevant molecule for immune defense mechanisms.

## 5. Related Mechanisms and Future Research Directions

### 5.1. From HHV-6 Childhood Infection to AD in Older Individuals—The Neuroinflammatory Hypothesis

An important aspect surrounding the causative link between HHV-6 and AD is the long period of time elapsed between initial infection, which almost always occurs during childhood, and the much later onset of AD that is generally encountered in older persons. Recent research suggests the implication of several micro-organisms in the pathogenesis of non-communicable diseases [58]. While the complete pathophysiological mechanism is unknown, it is clear that viral pathogens can be effective triggers that, in combination with a favorable genetic and phenotypic environment, lead to neurodegeneration [59]. Up to now, only common immunopathological mechanisms of chronic HHV-6 infection and neuroinflammation that lead to pathological conditions such as AD have been described. The pro-inflammatory cytokines are involved in both processes, with the pathologically-modified inflammatory cascades representing a core hypothesis for AD [60]. Irreversible CNS changes triggered by acute and chronic viral infections can be explained via the increased level of inflammatory factors that activate the pathological microglial phenotype, subsequently sustaining the vicious cycle of neuroinflammation.

Neuroinflammation is essential to our understanding of neurodegenerative diseases, with AD being no exception [61]. With regards to the triggering event, the common-sense belief is that any pathogenic element that enters the CNS is a potential inducer of neuroinflammation with possible chronic repercussions [62]. A recent study claims that an infection occurring in the periphery can favor the production of antigen-specific CD8 + memory T cells that can later reach the brain with subsequent consequences [63]. Moreover, given the impact of the intestinal microbiome in a variety of pathological conditions, it is suspected that an intestinal infection will stimulate the innate immune system of the brain via incompletely known humoral signaling pathways. At the same time, altered BBB and changes in the brain modulate the gut microbiome via the vagus nerve (afferent pathway), maintaining a state of chronic inflammation that can sustain the subsequent neurodegeneration encountered in AD [64]. It has been noted that the inflammatory theory does not contradict the other aforementioned AD hypotheses but rather acts as a link between the chronic inflammatory status and the intracellular and extracellular accumulation of misfolded proteins. Indeed, HHV-6 has been associated with the activation of glial cells. These cells subsequently produce pro-inflammatory factors, promoters of a chronic inflammatory state conducive to neurodegeneration [65]. Moreover, it seems that the action of HHV-6 is a direct one at the level of microglia, which induces activation and migration [66], being well known for its role in supporting neuroinflammation [67].

Viral CNS latency means more than just passive “hibernation” and waiting for reactivation in the case of host immunosuppression. The capability of viruses, including HHV-6, to integrate into the subtelomeric regions of host chromosomes has long been known, including the effects of genetic mutation on host DNA [68]. Moreover, in approximately 1% of the human population, chromosomally integrated HHV-6 has been reported to be integrated into gametes, thereby explaining the inheritance of HHV-6 [69]. However, it is not yet clear whether viral cantonment in the genetic material takes place in “hot-spots” where gene mutations could cause or facilitate the appearance of dementia (possibly also other neurological diseases) or whether there exist other alterations of specific mechanisms that explain the increased infection rate in certain CNS cells (mainly neurons and immune cells) [70]. Another study representing the first transcriptome sequencing study [71] proposed the association of seven genes (CTSS, SERPINA1, NPTX1, PTX3, CHI3L1, SERPINA3, and MX1) as “the missing link” to explain the correlation between HHV-6A infection and neurological diseases. Another study revealed that the IDO1 gene is another important gene shared by both AD and HHV6 host responses [72]. Indeed, indoleamine 2,3-dioxygenase (IDO1) is involved in the kynurenine pathway, which is important for neuroinflammation and neurodegeneration [73].

Substantial evidence linking childhood-onset neuroinflammatory systemic alterations to the onset of neurodegenerative disorders such as AD exists. It still has to be determined, however, which other genetic and epigenetic factors are involved in the pathogenesis of AD and the complex mechanisms via which sustaining a chronic inflammatory state in the early years of living can lead to degeneration in adult ages.

### 5.2. HHV-6 CNS versus Peripheral Infection

A very important aspect of HHV-6 infection, which is crucial to our understanding of the relationship between the virus and AD, deals with the question of how viral CNS penetration occurs. Like other neurotropic viruses, HHV-6 appears to reach brain parenchyma via the CNS barriers (the blood–brain barrier—BBB, the blood–CSF barrier located at the choroid plexus, and the meningeal barrier) and via the olfactory tract. A study by Harberts et al. [74] showed that HHV-6 is found in high concentrations in the olfactory bulb and in other areas closely related to the olfactory pathway, such as the limbic system, the hippocampus, and the medial temporal lobe. According to the authors, the nasal cavity can be considered a natural in vivo reservoir, as evidenced by the similar prevalence of HHV-6 DNA in the nasal cavity compared to saliva (which is known to be a reservoir for HHV-6 in sub-clinical, latent infection). In support of this argument is the similarity between olfactory ensheathing cells (OECs) and other glial cells (mainly astrocytes). OECs maintain an environment for viral replication, subsequently guiding the virus through the BBB, although it has not been demonstrated in vitro on cell cultures.

Next in our discussion of viral CNS penetration is the topic of hematogenous spread. The BBB, a unique structure in the human body [75], is a crucial structure in the maintenance of brain homeostasis [76]. Structural and functional dysfunctions of this structure may lead to many neurological disorders [77]. One of the BBB’s primary roles is to prevent the penetration of toxins and exogenous pathogens into the sensitive brain parenchyma. The understanding of the detailed mechanism of herpes viruses (including HHV-6) crossing of the BBB is still incompletely elucidated but remains a potential pathway for CNS invasion. A recent review summarizes the most relevant aspects that relate the BBB breakdown to a possible HHV-6 entry at the CNS level but theoretical data remain without experimental support up to present [78].

Aside from HHV-6 tropism for nervous tissue, the role of peripheral HHV-6 infection in AD pathogenesis needs further study. The dysregulation of the immune system at the CNS level has already been demonstrated to be one of the key processes involved in neurodegeneration [79]. Furthermore, recent research underlines the importance of crosstalk between immune processes that occur in both central and peripheral compartments [80]. Accordingly, the HHV-6 impact in the periphery might play a role in further immune dysfunction at the CNS level, favoring neurodegeneration. In this context, we mention the work of Carbone et al. [19], who report that peripheral blood leucocytes showed a significant association between HHV-6 DNA in the periphery and HHV-6 level in the brains of AD patients, thus strengthening the argument in favor of an association between AD and HHV-6. Regarding potential mechanisms, cytokines seem to be one of the key factors linking peripheral to central inflammation [81]. Studies examining the causality between peripheral and central inflammation are scarce. We mention the work of Bettcher et al. [82], who noted a strong association between CSF and plasma macrophage inflammatory protein 1β (MIP-1β) levels, both correlated with high levels of AD brain markers such as Aβ and p-Tau.

In addition, cellular immunity represented mainly by T-cells is also dysregulated in AD [83], and, on the other hand, viral pathogens such as HHV-6 additionally modulate the T cell phenotype in the periphery. Alongside T-cell pathological activation in the peripheral compartment, research highlighted the association of increased T cells at the CSF level in AD patients [84]. Other data pointed toward the association of pathological CD3+ T cells gathering in cerebral parenchyma with AD classical biomarkers such as Tau protein level [85]. However, the absence of amyloidogenic-related immune cell changes suggests the function of the immune system in neurodegeneration is far more complex than expected.

Finally, an intensely discussed hypothesis with a significant impact on many pathologies is the brain–gut microbiota axis. At present, there are only incipient human studies suggesting a weak association between specific types of gut bacteria (such as increased Proteobacteria or reduced Ruminococcus) and AD CSF markers [86]. Understanding the immunology and gut microbiota mechanisms that influence the brain’s normal function are mandatory for arriving at a conclusion. It is clear that the directionality and timing of the abovementioned mechanisms and their therapeutic potential are poorly understood; research on the central-to-peripheral HHV-6 infection crosstalk has to be done in the near future.

### 5.3. From Neuroprotection to Neuroinflammation—The Double-Edged Blade

A research direction worth mentioning is related to the physiological neuroprotective mechanisms that, in pathological conditions such as neuroinflammatory states, become “double-edged swords” and have rather more destructive than protective effects. Related closely to the HHV-6–AD infection association, a mechanism that may have both protective and destructive impacts depending on the cerebral microenvironmental conditions is miR-155 inhibition. On the one hand, miR-155 is linked to T cell dysregulation [87], another important feature of AD pathophysiology. Additionally, the suppression of miR-155 was demonstrated to promote the microglial switch from the M1 pro-inflammatory phenotype to the neuroprotective M2 phenotype [88]. On the other hand, data from earlier studies conducted on AD animal models might be confusing as the inhibition of miR-155 expression was shown to be correlated to increased Aβ plaque level at the cerebral level [56]. HHV-6 was demonstrated to reduce the miR-155 expression in different cells; thus, the inhibition of miR-155 may be a linking factor between HHV-6 infection and AD onset. Based on the available research, it cannot be concluded whether miR-155 inhibition or enhancement leads to the altered molecular and cellular pathways encountered in AD, but its modulatory effect is obvious.

## 6. Conclusions

Currently, there are no effective or curative treatment options for AD; thus, to be able to delay the onset and evolution of the disease, a better understanding of potential risk factors is needed. According to existing data, the causality and timing between HHV-6 infection and AD cannot be proven. However, research conducted on brain specimens and humans demonstrated an increased HHV-6 load at the CNS level, pointing towards a complex association that includes various intricate pathophysiological mechanisms.

There appears to be disagreement among study protocols and methodologies and this needs to be addressed as it could significantly impact our current knowledge and inform forthcoming research. Future work should be guided by an effort to address the apparently contradictory results, which in this case seem to arise from insufficiently sensitive HHV-6 detection methods coupled with the absence of a specific threshold and/or criteria related to the detection of HHV-6 with pathological significance [89].

Furthermore, and very importantly, future work should insist on the cellular and molecular pathways that directly and indirectly link chronic HHV-6 infection to neuroinflammatory/neurodegenerative processes considered hallmarks of AD.

Finally, the impact of antiviral therapy for HHV-6 infection and the development of AD is another interesting research direction, with no studies, to our knowledge, conducted to date.

## Figures and Tables

**Table 1:** The keywords used and the literature search strategy.

Search	Keywords
#1	“HHV-6” OR “HHV-6A” OR “HHV-6B” OR “Human herpesvirus
#2	“Alzheimer’s disease” OR “Alzheimer’s dementia” OR “dementia”
#3	#1 AND #2
**Final results**	
Identified records	PubMed (*n* = 44) Google Scholar (*n* = 639) Science Direct (*n* = 228)
Excluded records (duplicates, not eligible)	*n* = 891
Included records in the review	*n* = 20

## Data Availability

All data and materials supporting the results of the present study are available in the published article.
